# The Influence of Carbon-Carbon Multiple Bonds on the Solvolyses of Tertiary Alkyl Halides: a Grunwald-Winstein Analysis

**DOI:** 10.3390/ijms9091704

**Published:** 2008-09-04

**Authors:** Marina C. Reis, Ruben Elvas-Leitão, Filomena Martins

**Affiliations:** 1 Department of Chemistry and Biochemistry, Sciences Faculty, University of Lisbon, CQB, Campo Grande, 1749–016 Lisboa, Portugal. E-Mail: marina.reis@fc.ul.pt (M. R.); 2 Department of Chemical Engineering, Lisbon Institute of Engineering, IPL, R. Conselheiro Emídio Navarro, 1, 1959–007 Lisboa, Portugal. E-Mail: rleitao@deq.isel.ipl.pt (R. E-L.)

**Keywords:** Grunwald-Winstein Equation, Solvent effects, Carbon-carbon multiple bonds, Tertiary alkyl halides

## Abstract

The influence of carbon-carbon multiple bonds on the solvolyses of 3-chloro-3-methylbutyne (**1**), 2-chloro-2-phenylpropane (**2**), 2-bromo-2-methyl-1-phenylpropane (**3)**, 4-chloro-4-methyl-2-pentyne (**4**) and 2-chloro-2-methylbutane (**5**) is critically evaluated through the extended Grunwald-Winstein equation. Substrates **1**, **3** and **5** lead to correlations with unexpected negative sensitivity, *h*, to changes in the aromatic ring parameter, *I*. It is claimed that *I* is not a pure parameter, reflecting also some solvent nucleophilicity, *N*_OTs_, character. In substrates **2** and **4** the possibility of rearside solvation is reduced due to steric hindrance and/or cation stabilization and the best found correlations involve only the solvent ionizing power, *Y*, and *I.*

## 1. Introduction

The study of solvent effects in reactivity has been one of the cornerstones of physical organic chemistry, and still constitutes these days one of the most fascinating scientific challenges. The ongoing interest over these studies not any longer resides in the possibility of making predictions of rate constants for other solvents, but especially in its potential to assist us in the understanding of the true nature of substrate - solvent - solvent interactions, at a molecular level. In this context, there has been a continuing interest from the scientific community in the study of the solvolysis reactions of tertiary alkyl halides, which are still considered good model systems to monitor solvent and solvation effects [1–3]. One of the most used linear solvation energy relationships (LSER) to study solvent effects, in particular in aqueous organic media, has been the Grunwald-Winstein (G-W) equation, proposed for the first time in 1948 [4]. This equation, originally intended for use with S_N_1 (and E_1_) solvolyses, correlates the reaction rate, *k*, of an RX substrate in a given solvent with an empirical solvent parameter, *Y* (eq. 1):

(1) logk=mY+c

where *m* represents the substrate sensitivity towards changes in the solvent ionizing power, *Y*, and the independent parameter *c* is the substrate rate constant in a reference solvent, usually 80% ethanol/water (v/v). The *Y* scale was initially derived for the solvolyses of *t*-butyl chloride, but it has been shown that this compound’s solvolyses occur with a non-negligible nucleophilic character [2, 5–7]. For this reason, the most commonly used *Y* scales are now based on the solvolyses of 1- and 2-adamantyl derivatives (originating different *Y*_x_ scales depending on the leaving group X), since these substrates are unable of undergoing either elimination or rearside nucleophilic attack from the solvent (or at least are strongly impeded from doing so) and seem therefore more appropriate model substrates to define scales of solvent ionizing power [2, 8] (during the peer review process of this manuscript, one of the Editors kindly drew these authors’ attention to a very recent paper on development and applications of the G-W equation [9]; in the referred paper, a very exhaustive review on the establishment and development of the several scales used in G-W approaches is made.).

In order to be able to apply eq. 1 to substrates reacting with the involvement of some nucleophilic solvation contribution, the introduction of an additional term to eq. 1 to explicitly consider the solvent’s nucleophilicity, *N*, was proposed (eq. 2) [10–12]:

(2) logk=mY+lN+c

In this extended Grunwald-Winstein equation, *l* is the sensitivity of the substrate to changes in solvent nucleophilicity. Throughout the years a number of solvent nucleophilicity scales have been proposed but the most frequently used are the *N*_OTs_ scale based on methyl tosylate solvolysis [7,13–15] and the *N*_T_ scale based on the solvolysis of *S*-methyldibenzothiophenium ion [7, 14–16]. As Mayr *et al.* [14] pointed out, in spite of the good correlation of these two scales with each other, as well as with some other solvent nucleophilicity scales, there is a still a vivid ongoing discussion on the role of nucleophilic solvent participation (NSP) in solvolysis reactions [17, 18]. This is certainly related to the indiscriminate use of the terms nucleophilic solvent participation and nucleophilic solvation (NS) as recently claimed by Richard *et al.* and other authors [3, 19].

Equations (1) and (2) have been successfully applied to substrates with localized charges [20, 21]. However, when the substrate’s cationic charge becomes resonance delocalized, the solvent stabilization effect can no longer be accounted for solely by the solvent parameter *Y* [22, 23]. Kevill *et al.* have shown that the introduction of a new solvent parameter, which they named as “aromatic ring parameter”, *I*, based on the solvolyses of (*p*-methoxybenzyl)dimethylsulfonium ion and 1-adamantyldimethylsulfonium ion (as stated by Kevill and co-workers, the *I* scale is based on differences in the nature of the variations of rate constants of solvolysis of the *p*- (methoxybenzyl)dimethylsulfonium ion and of the 1-adamantyldimethylsulfonium ion (no aromatic ring) as the solvent composition is altered: *I* = log (*k*/*k*_0_)_p-MeOC6H4CH2SMe2_ -1.3 log (*k*/*k*_0_)_1-AdS_+_Me2_, where *k*_0_ is the rate of solvolysis in 80% ethanol [15]), corrected for the dispersion observed in Grunwald-Winstein plots for substrates with aromatic rings which could enter into conjugation with the developing positive charge at the α- carbon [15, 23]. These authors have thus proposed the inclusion of this new term in eq. (2) to comprise those situations for which there was cationic charge delocalization by resonance:

(3) logk=mY+lN+hI+c

*I* measures the ability of the solvent to stabilize the delocalized cationic charge and *h* represents the substrate’s sensitivity to changes in the aromatic ring parameter, *I*, and should be related to the degree of cationic charge delocalization into the ring. The greater the degree of charge delocalization, the more stable the carbocation and therefore the larger the magnitude of *h* [22].

It was often observed that a significant *hI* contribution was usually accompanied by a negligible *lN* contribution and eq. (3) appears sometimes in the following truncated form [23, 24]:

(4) logk=mY+hI+c

Kevill *et al.* have illustrated rather profusely the statistical improvements in the G-W correlations for the solvolyses of several (secondary and) tertiary benzylic derivatives as a result of the inclusion of the *hI* term [15, 25]. However, these correlations are, in general, statistically comparable to those obtained for the same substrates by an alternative approach using appropriate similarity model scales based on the solvolysis of benzylic derivatives (*Y*_BnX_, *Y*_BnOTs_), as proposed by Liu and co-workers [26]. In fact, both authors have put forward a set of serious arguments to demonstrate the goodness of their methodologies. It is not our intention to enter this discussion but rather call the attention for a few other puzzling results, in the same line as those obtained by Kevill’s group when they showed, rather unexpectedly, that the best correlations obtained for the solvolysis of 4-chloro-2,2,4,6,6-pentamethyl-heptane and 3,3-dimethyl-1-neopentylbutyl mesylate included significant *I* contributions, even though these compounds have no aromatic rings or other conjugate multiple bonds [24]. These authors justify these apparent awkward results by saying that “for lack of a better term, what (they) label as the aromatic ring parameter could well be governed to some degree by perturbations due to variations in ion-pair return”, since, as they claim, it is difficult to distinguish between ion-pair return and aromatic ring solvation effects [23]. Bentley *et al.* [27], further state that predictions of solvent effects on solvolytic reactions can be attained through eq. 5, if one makes the appropriate choice of the similarity model to use and that this approach accounts for solvation effects adjacent to the reaction center in systems involving alkyl, alkenyl, alkynyl and aryl groups in various aqueous binary mixtures.

(5) $$\text{log}k=Y_{sim}+c$$

However, these authors emphasize that the reliability of these predictions depend in a great extent on the choice of the model and that therefore the proliferation of *Y* scales is not a minor problem.

The motivation for the research here presented came from our interest on the solvolysis reactions of tertiary alkyl halides [3, 28]. In the context of our systematic work on solvent effects, we came across the need to isolate and rationalize certain solvent contributions and for that reason we had to select some particular substrates. Among the various LSER equations available in the literature, the methodology used within our group to evaluate the solvent’s involvement in these reactions has been the TAKA model equation [29]. However, the kinetics of the newly addressed substrates turned out to be extremely slow in some pure solvents preventing the use of this approach in an appropriate set of solvents, both in number and diversity. An alternative way to obtain rate constants in a reasonable time scale and also some information on the processes appeared to us to be the use of solvent mixtures and Grunwald-Winstein plots, which we have shown in previous work [30] to have unsuspected affinities with the TAKA approach.

Hence, in the present work the Grunwald-Winstein extended equation, eq. (3), or one of its truncated versions, was used to probe the influence of carbon-carbon multiple bonds associated to tertiary carbons on the reactivity of a number of alkyl halides, namely, 3-chloro-3-methylbutyne (**1**), 2-chloro-2-phenylpropane (**2**), 2-bromo-2-methyl-1-phenylpropane (**3**), and also 4-chloro-4-methyl-2-pentyne (**4**) [6, 27].

For comparative purposes, we have also applied eq. 3 to a substrate already studied by Liu *et al.* [20] for which there is no possibility of charge delocalization, namely 2-chloro-2-methylbutane (**5**).

## 2. Results and Discussion

Rate constants for the solvolyses of substrates **1** to **3** were determined at 25.00 °C in several pure and mixed solvent mixtures (Table 1). Table 1 also presents rate constants from literature for the solvolyses of substrates **4** [6, 27] and **5** [20], and of substrate **2** in 80% acetone/water [31] and **3** in 40% trifluoroethanol/ethanol [32], as well as reported *Y*_Br_, *Y*_Cl_, *N*_OTS_ and *I* values for each solvent (for references, see footnote on Table 1).

### 2.1. Correlations of log k with solvent ionizing power as measured by Y_X_

**Figure 1.**
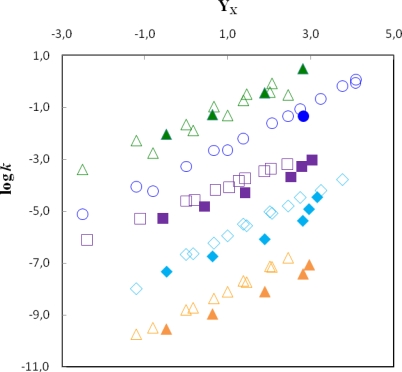
log *k vs. Y*_Cl_ for the solvolyses of **1** ( 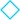), **2** ( 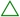), **4** ( 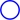) and **5** ( 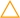) at 25,00 °C and of log *k vs. Y*_Br_ for the solvolyses of **3** ( 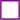) at 25,00 °C. The filled symbols correspond to the solvolyses of **1** ( 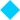), **2** ( 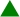), **3** ( 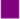), **4** ( 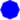) and **5** ( 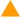) in TFE and its mixtures. The points for **3** and **5** are shifted downwards by 0.5 and 4 units, respectively, for clarity.

**Figure 2.**
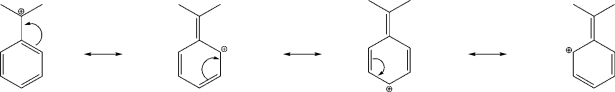
Resonance structures for 2-phenylprop-2-ylium ion.

**Figure 3.**
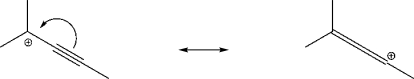
Resonance structures for 2-methylpent-3-yn-2-ylium ion.

To help visualizing the relative reactivity of substrates **1** to **5,** we plotted log *k vs. Y*_X_ (X=Cl or Br) - Figure 1. The significant differences observed in these substrates’ reactivity might be attributed to different degrees of carbocation stabilization in the five compounds. In substrates **2** and **4** positive charge can be stabilized by resonance, as illustrated in Figures 2 and 3, thus leading to a higher solvolysis rate. This type of stabilization effect is however precluded for substrates **1** and **3**, in the first case because the terminal carbon cannot accommodate the positive charge and in the latter case because the introduction of a CH_2_ group attached to the aromatic ring turns unfeasible any conjugation with the developing carbocationic center. Compound **5**, as mentioned above, is unable to experience any charge delocalization.

Figure 1 also shows evident downward deviations for substrates **1**, **3** and **5** in the weakly nucleophilic solvents, *i.e.*, TFE and its mixtures. This fact suggests, by comparison with the model adamantyl compounds, the intervention of nucleophilic solvation in the stabilization of the transition state of these solvolyses, justifying therefore a higher reactivity, in the cases of EtOH, MeOH, their aqueous mixtures and aqueous acetone, which is in line with other authors’ findings [6, 20, 21, 24]. As Kevill [15, 24] and Bentley [6] have pointed out, an increase in steric hindrance or in cation stabilization should reduce these deviations by diminishing the possibility of rearside nucleophilic solvation, which is observed in the set of compounds studied in this work for substrates **2** and **4** (and also for substrate **3** when compared to **1** and **5**).

### 2.2. Correlations of log k with G-W parameters

Rate data in Table 1 were analyzed by using the extended G-W equation, eq. 3 or one of its truncated forms. The *m*, *l*, *h* and *c* values are summarized in Table 2 together with the associated standard errors. Several statistical criteria, such as the standard deviation of the fit, *sd*_fit_, the adjusted determination coefficient, *R*^2^_adj_, the Fisher’s *F* value and the significance level (*SL*) of each regression parameter (parameters are considered significant if *SL* > 95%) were computed for each regression. Intercorrelations among solvent parameters were tested in each case and were found to be negligible (*R*^2^ < 0.3).

In the study of solvent effects through multiparametric approaches, including Grunwald-Winstein analyses, one should be particularly attentive to the number and variety of solvents used and to the judicious use of statistical criteria, among other aspects, to guarantee a reliable interpretation of results and assure a correct evaluation of each solvent contribution to the process under study. Equally important is, however, the understanding and ascription of a clear physicochemical meaning to each parameter.

A quick overview of Table 2 shows that the number of solvents used for each substrate was always well over the required number to apply a three parameter fitting equation and, in general, the data sets present an adequate degree of solvents’ variability. Yet, correlations were not always performed, for each compound, on the same number of solvents. Although it is desirable to do so for comparative purposes, a multiparametric analysis advises the maximization of the number of solvents used in order to prevent chance correlations. On the other hand, given the nature of the solvents studied, the total variability is not considerably disturbed by small differences in the number of solvents, provided the fluorinated alcohols are maintained in the set. Substrate **4** is, in this context, a special case because to our knowledge and quite unfortunately there is only one literature value for this compound in fluor-alcohol containing solvents (97% TFE). Therefore, for this substrate, we cannot say that solvents’ variability is strictly the same as for the other substrates. However, the use of the adjusted determination coefficient, *R*^2^_adj_, permits a direct comparison among regressions involving a dissimilar number of points as this quantity corrects *R*^2^ for different degrees of freedom. For checking purposes, correlations for compounds **1** to **3** and **5** were also performed on the 14 common solvents and both parameters’ coefficients and associated errors remained essentially unchanged.

A closer inspection of Table 2 shows that all *c* values, within experimental (see below) and calculation uncertainties, coincide with experimental log *k* values reported in Table 1 for the reference solvent (80% EtOH/ water), which is a good indication of the regressions’ quality since this solvent mixture was always used as part of the solvents’ set.

For substrates **1**, **3** and **5**, the best correlations in terms of all listed statistical criteria involve the three terms, *mY*, *lN*_OTs_ and *hI* (eq. 3), being *m* approximately equal in **1** and **5** and rather low in **3**. Following Takeuchi’s interpretation [21], a lower *m* value in a more reactive compound (higher *c*) could be ascribed to an earlier transition state and, therefore, to a lesser sensitivity of **3** to the ionizing power of the solvent. For these three compounds the *N*_OTs_ term, although small, is statistically significant, corroborating the observed behavior in Figure 1 and the suggestion of some nucleophilic solvation contribution. The magnitude of this influence was expected to be smaller for compound **3** due to a lesser accessibility of the solvent to the developing carbocationic center because of steric constraints caused by the bulky aryl group and larger for compound **1** which cannot stabilize the developing positive charge and has no steric constrictions. In fact, if we compare the magnitude of *l* for the two term equation (eq. 2) for the three compounds we see that *l* values increase in the order **3** (*l* = 0.26 ± 0.03) < **5** (*l* = 0.32 ± 0.05) < **1** (*l* = 0.39 ± 0.04). Further, we would anticipate a non-significant *I* term for these substrates, since there is no possibility of charge delocalization. So, finding eq. 3 as the best equation in these three cases, and moreover, obtaining negative *h* values with a statistical significance higher than 97%, was rather surprising (In the recent review paper mentioned above on page 2, lines 22–25, negative *h* values are also reported but in correlations for the capture by solvent of three extensively charge delocalized carbocations, namely (4-methoxyphenyl)phenylmethyl-, (4-methoxyphenyl)(4-methylphenyl)methyl- and di(4-methoxyphenyl)methyl cations. This negative contribution was claimed to be expected when capture rather than release of this type of cations is involved [9]). This fact leads us to question the grounds in which the *I* scale has been established. If *N*_OTs_ is not exactly the same for the two model compounds used to establish the scale (the *p*-(methoxybenzyl)-dimethylsulfonium ion and the 1-adamantyldimethylsulfonium ion), then the *I* parameter, as it has been used, is not a pure parameter, and incorporates a certain *N*_OTs_ character. This would thus be responsible for a downward deviation of the *h* values, pushing them away from zero. This effect would explain the tendency observed in the relative magnitude of *l* when we apply eq. 2 and which is not visible when we apply eq. 3.

For compound **2** the application of eq. 2 leads to a negative *l* value which becomes small and positive, when full eq. 3 is considered but with a statistical significance at a much lower level than the chosen 95% value. This tendency was already observed by Kevill *et al.*, for instance, for a set of benzylic tosylates [15] and other benzylic derivatives [23]. Clearly, the best found correlation corresponds to eq. 4, involving both *mY* and *hI*. This result was anticipated because of steric hindrance which renders difficult the solvent’s access to the incipient tertiary carbocation and also due to the possibility of charge delocalization by resonance into the ring. The *h* value found compares nicely with the literature value of 0.58 ± 0.28 [26] obtained for a smaller number of solvents.

Substrate **4** was previously studied by Bentley *et al.* [6, 27] and also considered by Kevill *et al.* [23] in a comparative study of solvolysis in 16 solvents (including no fluorinated alcohols) of allyl and propargyl derivatives with and without the aromatic ring parameter. In Table 2 we show the application of eqs. 1 to 4 to this substrate, with and without the inclusion of the point relative to 97% TFE. If this solvent is included, the best correlation is achieved by applying eq. 3, which leads to statistical significant *l* and *h* terms. However, using a unique value for the fluor alcohol-containing solvents (which, moreover, have in general large positive *I* values and enhance solvolysis rates by strongly stabilizing ring delocalized cationic charges [22]) does not seem either sufficient to guarantee a reasonable solvents’ diversity or enough to judge the influence of these solvents on the aromatic ring parameter contribution. We have, instead, chose to show the results for this substrate in a set of 14 solvents, *i.e.*, excluding the 97% TFE point and with this procedure have avoided using an evident leverage point. In doing so, we arrive to eq. 4 as the best correlation, involving only the *mY* and *hI* terms, just like Kevill *et al.* in the referred work. In fact, the methyl group attached to the triple bond accelerates the rate of solvolysis in 80% EtOH (see *c*) approximately 2500-fold when compared to H (substrate **1**), possibly due to a combination of conjugative and hyperconjugative effects, as already pointed out by Bentley *et al.* [27]. On the other hand, the values for *m* and *h* are quite similar for substrates **4** and **2**.

Finally, the comparison of *c* values for substrates **2** and **3** discloses a different behavior of the phenyl ring in both substrates. In **3** the phenyl group is operating solely as an electron-withdrawing group, decreasing therefore its reactivity in the reference solvent. On the contrary, in **2** the magnitude of *c* clearly shows that positive charge stabilization by resonance into the ring successfully overcomes the electron-withdrawing effect.

## 3. Experimental Section

2-Chloro-2phenylpropane and 2-bromo-2-methyl-1-phenylpropane were synthesized by standard methods [3, 34]. 3-Chloro-3-methylbutyne was obtained commercially. The three compounds were purified by column chromatography (Silica gel 60) and their purity was subsequently assessed by NMR (^1^H-NMR spectra were recorded at 400 MHz in CDCl_3_). The substrate concentration used in the kinetic experiments was 0.01 mol dm^−3^. Solvents were obtained commercially from Aldrich and Riedel-de Häen with purities above 98%. Kinetic measurements were made at 25.00 °C using an automated conductance bridge. Temperature control was always better than 0.01 °C. Reactions were followed to at least 90% of the apparent plateau. Conductance values were assumed to be linearly related to the acid concentration formed during the reaction. *k* values were derived using an Excel spreadsheet previously designed for this purpose [35]. Mean *k* values resulted from at least three different runs and showed a standard deviation better than 4%.

## 4. Conclusions

In this paper we report a comparative study of the influence of carbon-carbon multiple bonds on the solvolyses of 5 tertiary alkyl halides by using the Grunwald-Winstein extended equation. A careful correlation analysis was undertaken which lead to some unexpected results, that is, a negative coefficient associated to the “aromatic ring parameter”, *I*, for substrates without possibility of any charge delocalization, namely substrates, **1**, **3** and **5**. The reason for this is claimed to be related to the possibility of a non-negligible *N*_OTs_ character in the *I* parameter, due to the absence of any correction for differences in *N*_OTs_ in the two model compounds used to establish the *I* scale. On the other hand, the best correlations found for substrates **2** and **4** show that the solvent nucleophilicity contribution, as measured by *N*_OTs_, is not statistically significant, in line with the increase in steric hindrance and/or charge stabilization in these compounds. Also, the analysis of *c* values gives evidence for a different behavior of the phenyl group in substrates **2** and **3**. While in **3** the ring is acting only as an electron-withdrawing group, diminishing the intrinsic reactivity of the substrate, in **2** the stabilization of the positive charge through resonance is the prevailing effect, leading therefore to a higher *c* value.

Further studies of solvent effects on the reactions of other tertiary substrates with several degrees of branching and involving different types of carbon-carbon multiple bonds will certainly contribute to shed more light onto the understanding of the true role of the various solvent parameters involved in these G-W equations.

## Figures and Tables

**Scheme 1.**
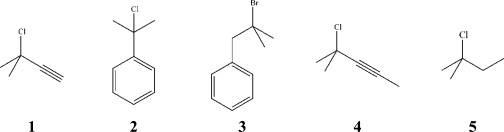
Compounds analyzed in this study.

**Table 1.** log *k* values and solvent parameters for the solvolyses of substrates **1** to **5**, at 25.00 °C.

Solvent^a^	*Y*_Br_^b^	*Y*_Cl_^b^	*N*_OTs_^b^	*I*^c^	**log *k***				
					1	2^d^	3	4^e^	5^f^
80%Ac	−0.7	−0.8	−0.42	−0.23	—	−2.75^g^	—	−4.22	−5.49
70%Ac	0.2	0.17	−0.42	−0.29	−6.63	−1.88	− 4.08	—	−4.72
60%Ac	1.03	1.00	−0.41	−0.28	−5.94	−1.30	−3.58	−2.64	−4.10
40%Ac	2.44	2.46	−0.38	−0.35	−4.79	−0.51	−2.69	−1.34	−2.79
20%Ac	3.66	3.77	−0.38	−0.40	−3.77	—	—	−0.17	—
100%Me	−1.12	−1.20	−0.04	0.41	−7.98	−2.27	−4.78	−4.06	−5.74
80%Me	0.70	0.67	−0.05	0.14	−6.22	−0.96	−3.68	−2.66	−4.36
70%Me	1.42	1.46	−0.08	0.05	−5.55	−0.48	−3.24	—	−3.73
60%Me	2.04	2.07	−0.13	−0.02	−5.07	−0.07	−2.86	−1.61	−3.14
40%Me	3.14	3.25	−0.21	−0.13	−4.19	—	—	−0.67	—
20%Me	3.94	4.10	−0.35	−0.26	—	—	—	0.08	—
100%Et	−2.40	−2.50	0.00	0.20	—	−3.38	−5.60	−5.12	—
**80%Et**	**0.00**	**0.00**	**0.00**	**0.00**	**−6.66**	−**1.65**	**−4.11**	**−3.27**	**−4.80**
60%Et	1.26	1.38	−0.08	−0.15	−5.49	−0.72	−3.33	−2.20	−3.68
50%Et	1.88	2.02	−0.09	−0.20	−5.01	−0.40	−2.96	—	−3.12
40%Et	2.62	2.75	−0.23	−0.24	−4.46	—	—	−1.06	—
20%Et	3.92	4.09	−0.34	−0.33	—	—	—	−0.04	—
TFE	2.53	2.81^h^	−3.07	0.37	−5.37	0.50	−3.18	—	−3.42
97% TFE	2.53	2.83	−2.79	0.49	—	—	—	−1.33	—
70% TFE	2.79	2.96	−1.2	0.25	−4.92	—	−2.78	—	−3.07
50% TFE	3.04	3.16	−0.93	0.09	−4.46	—	−2.52	—	—
80TFE20Et	1.42^i^	1.89^h^	−1.72	0.52	−6.08	−0.43	−3.79	—	−4.10
60TFE40Et	0.44^i^	0.63^h^	−1.01	0.59	−6.74	−1.27	−4.33	—	−4.96
40TFE60Et	−0.57	−0.48^h^	−0.55	0.43	−7.33	−2.03	−4.79^j^	—	−5.54
97% HFIP	4.51	5.08	−4.27	0.73	—	—	—	—	−1.88
Water	4.44	4.57	−0.44	−0.45	−3.09	—	—	—	—

a Ac, Me, Et, TFE refer to acetone, methanol, ethanol and 2,2,2-trifluoroethanol, respectively. The number indicates the volume percent of the particular solvent in the mixture, except for the mixtures TFE-water in which the number refers to weight percent.

b From ref. [8].

c From ref. [23].

d L. Moreira unpublished work. Higher log *k* values were obtained by extrapolation, from appropriate mixtures not used in the correlations.

e From refs. [6] and [27].

f From ref. [20].

g From ref. [31].

h From ref. [33].

i Values kindly provided by K-T Liu.

j From ref. [32].

**Table 2.** Correlations of log *k vs.* various combinations of *Y*, *N*_OTs_ and *I* parameters for the solvolyses of **1** to **5** at 25.00 °C.

*Substrate*	*m*±*s*(*m*) (%*SL*)^a^	*l*±*s*(*l*) (%*SL*)^a^	*h*±*s*(*h*) (%*SL*)^a^	*c*±*s*(*c*) (%*SL*)^a^	*sd*_fit_^b^	*R*^2^_adj_^c^	*F*^d^	*n*^e^	*eq.*
1	0.79 ± 0.05 100%	—	—	−6.88 ± 0.11 100%	0.315	0.934	271		1
1	0.84 ± 0.02 100%	0.39 ± 0.04 100%	—	−6.75 ± 0.05 100%	0.129	0.989	854	20	2
1	0.70 ± 0.03 100%	—	−0.92 ± 0.13 100%	−6.71 ± 0.06	0.164	0.982	521		4
1	**0.78 ± 0.03** **100%**	**0.26 ± 0.04** **100%**	**−0.45 ± 0.10** **100%**	**−6.71 ± 0.03** **100%**	**0.088**	**0.995**	**1240**		**3**
2	0.69 ± 0.04 100%	—	—	−1.72 ± 0.07 100%	0.264	0.936	219		1
2	0.68 ± 0.05 100%	−0.04 ± 0.10 33%	—	−1.74 ± 0.08 100%	0.272	0.932	104	16	2
2	**0.71 ± 0.04** **100%**	—	**0.52 ± 0.18** **99%**	**−1.78 ± 0.06** **100%**	**0.214**	**0.958**	**172**		4
2	0.75 ± 0.05 100%	0.14±0.09 86%	0.72 ± 0.21 99%	−1.75 ± 0.06 100%	0.203	0.962	128		3
3	0.57 ± 0.04 100%	—	—	−4.22 ± 0.06 100%	0.217	0.936	236		1
3	0.63 ± 0.02 100%	0.26 ± 0.03 100%	—	−4.14 ± 0.03 100%	0.094	0.988	654	17	2
3	0.54 ± 0.02 100%	—	−0.57 ± 0.12 100%	−4.15 ± 0.04 100%	0.139	0.974	298		4
3	**0.60 ± 0.02** **100%**	**0.20 ± 0.04** **100%**	**−0.24 ± 0.09** **97%**	**−4.13 ± 0.03** **100%**	**0.080**	**0.991**	**607**		**3**
4	0.79 ± 0.02 100%	—	—	−3.28 ± 0.05 100%	0.150	0.991	1622		1
4	0.80 ± 0.02 100%	—	—	−3.27 ± 0.04 100%	0.128	0.994	2173^f^		1
4	0.81 ± 0.02 100%	0.15 ± 0.05 99%	—	−3.25 ± 0.04 100%	0.116	0.995	1357		2
4	0.82 ± 0.01 100%	0.63 ± 0.20 99%	—	−3.17 ± 0.04 100%	0.097	0.997	1920^f^	15	2
4	0.79 ± 0.02 100%	—	−0.01 ± 0.17 6%	−3.28 ± 0.05 100%	0.156	0.991	749		4
4	**0.85 ± 0.02** **100%**	—	**0.58 ± 0.15** **100%**	**−3.27 ± 0.03** **100%**	**0.087**	**0.997**	**2356**^f^		4
4	0.84 ± 0.02 100%	0.25 ± 0.05 100%	0.42 ± 0.12 99%	−3.24 ± 0.03 100%	0.084	0.997	1709		3
4	0.84 ± 0.05 100%	0.24 ± 0.28 58%	0.42 ± 0.24 89%	−3.24 ± 0.05 100%	0.088	0.997	1532^f^		3
5	0.65 ±0.05 100%	—	—	−4.89 ± 0.10 100%	0.324	0.910	163		1
5	0.83 ±0.04 100%	0.32 ± 0.05 100%	—	−4.85 ± 0.05 100%	0.165	0.977	335	17	2
5	0.69 ± 0.03 100%	—	−0.79 ± 0.14 100%	−4.85 ± 0.06 100%	0.180	0.972	279		4
5	**0.79 ± 0.03** **100%**	**0.20 ± 0.05** **100%**	**−0.44 ± 0.13** **100%**	**−4.84 ± 0.04** **100%**	**0.124**	**0.987**	**403**		**3**

a Significance level.

b Standard deviation of the fit.

c Adjusted determination coefficient.

d F statistics.

e Number of solvents included in the correlation.

f Correlation performed on 14 solvents without the value for 97% TFE.
